# Patient-oriented simulation based on Monte Carlo algorithm by using MRI data

**DOI:** 10.1186/1475-925X-11-21

**Published:** 2012-04-17

**Authors:** Ching-Cheng Chuang, Yu-Tzu Lee, Chung-Ming Chen, Yao-Sheng Hsieh, Tsan-Chi Liu, Chia-Wei Sun

**Affiliations:** 1 Institute of Biomedical Engineering and National Taiwan University Molecular Imaging Center, National Taiwan University, Taipei, Taiwan, Republic of China; 2 Biophotonics and Molecular Imaging Research Center, Institute of Biophotonics, and Biomedical Optical Imaging Lab, National Yang-Ming University, Taipei, Taiwan, Republic of China; 3 Department of Photonics, National Chiao Tung University, Hsinchu, Taiwan, Republic of China

**Keywords:** Patient-oriented simulation, Time-resolved Monte Carlo, Brain modeling, Spatial sensitivity profile

## Abstract

**Background:**

Although Monte Carlo simulations of light propagation in full segmented three-dimensional MRI based anatomical models of the human head have been reported in many articles. To our knowledge, there is no patient-oriented simulation for individualized calibration with NIRS measurement. Thus, we offer an approach for brain modeling based on image segmentation process with *in vivo *MRI T1 three-dimensional image to investigate the individualized calibration for NIRS measurement with Monte Carlo simulation.

**Methods:**

In this study, an individualized brain is modeled based on *in vivo *MRI 3D image as five layers structure. The behavior of photon migration was studied for this individualized brain detections based on three-dimensional time-resolved Monte Carlo algorithm. During the Monte Carlo iteration, all photon paths were traced with various source-detector separations for characterization of brain structure to provide helpful information for individualized design of NIRS system.

**Results:**

Our results indicate that the patient-oriented simulation can provide significant characteristics on the optimal choice of source-detector separation within 3.3 cm of individualized design in this case. Significant distortions were observed around the cerebral cortex folding. The spatial sensitivity profile penetrated deeper to the brain in the case of expanded CSF. This finding suggests that the optical method may provide not only functional signal from brain activation but also structural information of brain atrophy with the expanded CSF layer. The proposed modeling method also provides multi-wavelength for NIRS simulation to approach the practical NIRS measurement.

**Conclusions:**

In this study, the three-dimensional time-resolved brain modeling method approaches the realistic human brain that provides useful information for NIRS systematic design and calibration for individualized case with prior MRI data.

## Background

Near-infrared spectroscopy (NIRS) is a promising non-invasive brain imaging technique with a higher sampling rate than positron emission tomography (PET)/functional magnetic resonance imaging (fMRI) and a more precise and localized spatial resolution than Electroencephalography (EEG)/Magnetoencephalography (MEG). The NIRS technique provides information about the slow signal (i.e., hemoglobin response) and fast signal (i.e., neuronal activation) [1-5]. This optical method permitted several benefits as non-invasive, less expensive, non-ionizing radiation imaging, real-time measurement, compact implementation, long time monitoring and easy operation with high time resolution and adequate spatial resolution for continuously recording oxy- and deoxy-hemoglobin changes of brain. Also, NIRS offers a more comprehensive measurement of brain activity than blood-oxygenation-level-dependent (BOLD) fMRI.

Functional near-infrared brain imaging is achieved with the backscattering light detection by using source-detector pairs on the surface of human head [5-7]. For NIRS implementation, there are several issues that including signal-to-noise ratio evaluation, optimal choice of source-detector separation, the brain structural effects on light propagation and the brain volume sampled remain to be fully understood well. Therefore, the simulation approach is important for characterization of photon migration in human brain with various source-detector separations to provide helpful information for individualized design of NIRS system [8-10].

In the most previous studies, the simulation results were generally obtained by semi-infinity five-layer structure [11-22] or two-dimensional head model with a MRI slice [13,18]. Naturally, the three-dimensional brain structure modeling by utilizing *in vivo *MRI data provides a realistic phenomenon of photon migration dynamics. However, there is no detail description for efficient and systematic modeling method of Monte Carlo algorithm with three-dimensional anatomical MRI data [14,15,17,19]. Additionally, the three-dimensional model which faithfully represents the realistic human head from MRI data depends on image processing.

Therefore, we offer a systematic approach for 3D brain modeling based on image segmentation process with *in vivo *MRI T1 three-dimensional image. For investigation of individualized difference in brain structure with NIRS, an adult volunteer was modeling to implement Monte Carlo simulated with various source-detector separations. According to previously studies, the light guiding effect occurred in the CSF layer of human brain. The presence of a relatively clear layer such as CSF that has both low scattering and absorption coefficients has been shown especially to alter the light propagation in the head [12,16,19,23,24]. This phenomenon cannot be portrayed by diffusion approximation method because the CSF reveals low scattering property [25] but it can be observed in the Monte Carlo simulation. Accordingly, the result indicates the advantage of the Monte Carlo method for NIRS modeling. Besides, the NIRS system typically applies multi-wavelength sources to detect the concentration changes of oxy- and deoxy-hemoglobin such as 690 nm, 780 nm and 830 nm. Therefore, this study offers a NIRS simulation method for understanding photon migration dynamics in human brain by using three-dimensional MRI data with multi-wavelength illumination.

## Methods

### Three dimensional brain MRI T1 data processing

**Figure 1:**
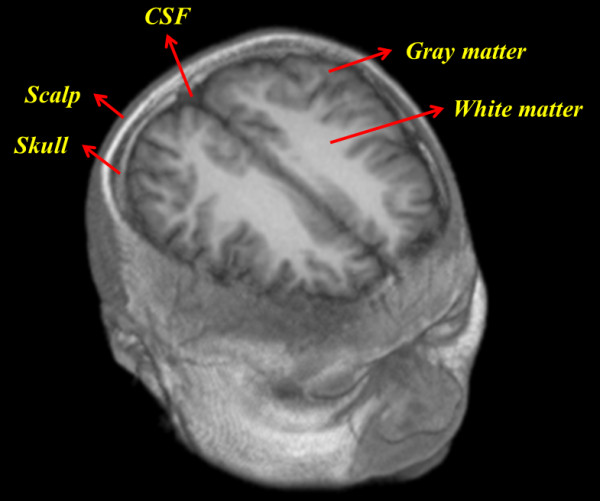
**Three dimensional *in vivo *MRI T1 brain image**. In the simulation, the three-dimensional MRI T1 brain image was considered with five layers as scalp, skull, cerebral spinal fluid (CSF), gray matter and white matter. The schematic diagram shows the anatomical structure of the human head.

Figure 1 shows an *in vivo *MRI T1 image of human brain with five layers that assigned as scalp, skull, cerebral spinal fluid (CSF), gray matter and white matter, respectively. The three-dimensional brain image contains 256 × 256 × 92 voxels and each voxel size is 1 × 1 × 1 mm^3^.

**Figure 2:**
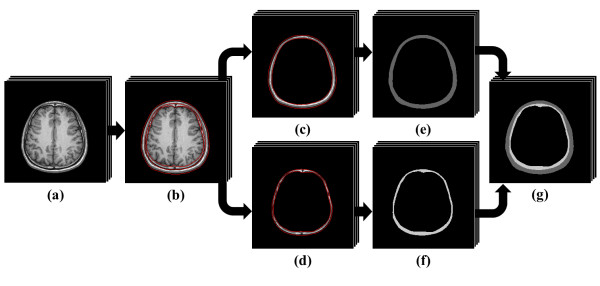
**Segmentation of scalp and skull layer**. This figure shows the segmentation process of scalp and skull: (a) two-dimensional anatomical MRI images, (b) contours segmentation with level set operator, (c) boundaries of the scalp and (d) skull layer, and (e), (f) segmentation with region growing approach (scalp = 1, skull = 2), (g) the two layers modeling of the scalp and skull.

Segmentation methods are important technique used in image processing to identify the objects in the image. For segmentation of brain layers, the image process includes two steps: 1) to segment the scalp and skull by level set method and region growing approach and 2) to segment the CSF, gray matter and white matter by using probabilistic framework segmentation. Figure 2 demonstrates the contour segmentation of scalp and skull that was achieved with level set and region growing operator. The level set method is a numerical technique for tracking interfaces and shapes. The advantage of the level set method are that it is implicit, parameter free, provides a direct way to estimate the geometric properties of the evolving structure, can change the topology and is intrinsic [26-29].

In the Figure 2(a), as could be expected, there are holes (boundary gaps) in the edges of skull layer, so simply coloring and parametric form of deformable models between the edges would not work. In contrast, level sets are designed to handle topological changes naturally. However, unlike the parametric form, they are not robust to boundary gaps and suffer from several other deficiencies as well. Thus, the level set method is suitable to segment the contour of scalp and skull from MRI data. In two dimensions, the level set method amounts to representing a closed curve *Γ *using a level set function *ø *(t, x, y). The closed curve *Γ *is represented as the zero level set of *ø *(t, x, y) by [29]

(1) Γ(t)={(x,y)|ø(t, x, y)=0}

If the curve *Γ*(*t*) moves in the normal direction with a speed function *F*, then the level set function *ø *satisfies the level set equation that can be written in the following general form:

(2) $$\frac{\partial ø}{\partial t}+F\left|\nabla ø\right|=0$$

To avoid the problem of further computation highly inaccurate with develop shocks, very sharp or flat shape during the evolution. The level set function *ø *have to reshape (also call re-initialize) to be a signed distance function periodically during the evolution. The variational level set formulation which keeps as an approximate signed distance function has been proposed that can be easily implemented by simple finite difference scheme, without the need of re-initialization as following the formula:

(3) $$\varepsilon \left(ø\right)\;=\;\mu \text{P}\;\left(ø\right)\;+\varepsilon_{m\;}\left(ø\right)$$

where *μ *> 0 is a parameter controlling the effect of penalizing the deviation of *ø *from a signed distance function; P(*ø*) is considered as the internal energy, defined within the curve, are designed to keep the model smooth during the deformation process. Consider a unit circle Ω⊂ℜ^2 ^that can be written as:

(4) $$\text{P}\;\left(ø\right)\;=\;\int_{\text{Ω}}\frac{1}{2}\;{\left(\left|\nabla ø\right|\;-\;1\right)}^{2}\;dxdy$$

while the ε_m _(*ø*) is considered as the external energy which are computed from the underlying image data, are defined to move the model toward an object boundary or other desired features within the image. The formula of external energy can be written as:

(5) $$\varepsilon_{m}\;\left(ø\right)\;=\;\varepsilon_{g,\lambda ,v}\;\left(ø\right)\;=\;\lambda L_{g}\;\left(ø\right)\;+\;v{\text{A}}_{g}\;\left(ø\right)$$

where λ > 0 and ν are constants; *L_g_*(*ø*) is the length of the zero level curve of *ø *and A*_g_*(*ø*) is introduced to speed up curve evolution of level set function that are defined as:

(6) $$L_{g}\;\left(ø\right)\;=\;\underset{\text{Ω}}{\int }g\delta \left(ø\right)\;\left|\nabla ø\right|\;dxdy$$

(7) $$A_{g}\;\left(ø\right)\;=\;\underset{\text{Ω}}{\int }gH\;\left(-ø\right)dxdy$$

where *δ *is the univariae Dirac function, and *H *is the Heaviside function; *g *is the edge indicator function defined by

(8) $$g\;=\;\frac{1}{1\;+\;{\left|\nabla G_{\sigma }\;*\;I\right|}^{2}}$$

where *G*_*α *_is the Gaussian kernel with standard deviation α and *I *is an image.

Figure 2(b) shows the results obtained using level sets to segment scalp and skull from the background. To obtain the outer boundary of the scalp, we started with an initial level set at the boundary of the image. To obtain the structures of skull on the inside of the brain, we started with an initial level set that was a closed curve around a point on the inside. This curve evolved to identify the boundaries of skull inside the brain. The contours generated by the level sets are closed contours.

After image segmentation by utilizing level set method, the contours of scalp and skull were segmented as shown in Figure 2(c) and 2(d). The region growing approach was then adopted to segment the two connected layers in binary image. The basic idea of region growing was starting with seeds. The grow regions from corresponding seeds revealed similar properties with their neighboring pixels [30,31]. According to the result of region growing segmentation, the scalp and skull layers were distinguished and marked as type 1 and 2 in simulation (Figure 2(e) and 2(f)). Figure 2(g) shows the two layers modeling of the scalp and skull.

After scalp and skull labeling, the probabilistic framework was then applied to classify CSF, gray matter and white matter layers with unified segmentation, which was performed by fitting a mixture of Gaussians (MOG) model with prior information of deformable tissue probability maps [32]. The MOG model can be described by the probability density of intensity *y*_i _and *k*_th _Gaussian distribution with mean *μ*_k _and variance *σ*_k_^2 ^as

(9) $$P\left(y_{i}\;|\;c_{i}\;=\;k,\mu_{k},\sigma_{k}\right)\;=\;\frac{1}{\sqrt{2\pi \sigma_{k}^{2}}}\text{exp}\left(-\frac{{\left(y_{i}\;-\;\mu_{k}\right)}^{2}}{2\sigma_{k}^{2}}\right)$$

**Figure 3:**
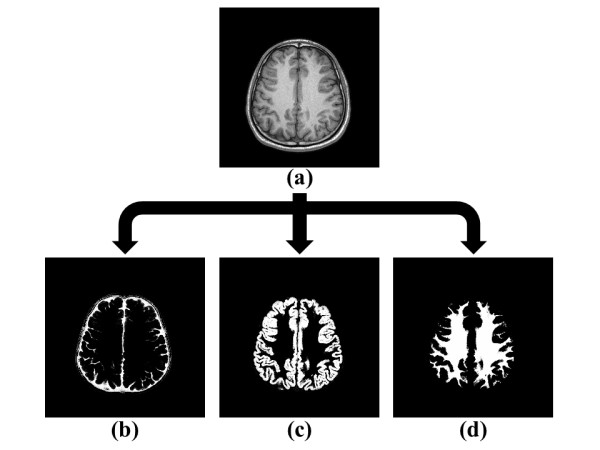
**Segmentation of CSF, gray matter and white matter**. Image process by using unified segmentation. (a) MRI data; (b) segmented CSF, (c) segmented gray matter, and (d) segmented white matter.

The Gaussian function indicated the probability distribution of brain tissues. The bias correction and image registration were included within the unified segmentation approach. Figure 3 shows the probability distributions of CSF, gray matter and white matter by unified segmentation from *in vivo *MRI data.

**Figure 4:**
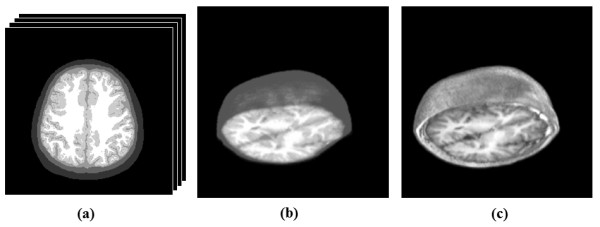
**The three-dimensional brain model with five layers**. Five layers three-dimensional brain structure in Monte Carlo simulation: (a) one slice of brain model with five layers, (b) reconstructed three dimensional brain model, (c) original three dimensional MRI data.

According to the mapping of probability distribution, each image pixel would be sorted to CSF, gray matter, or white matter by calculated maximum probability of tissue type. The layers of CSF, gray matter and white matter were assigned as type 3, 4 and 5 in simulation. Figure 4(a) shows the five-layer brain structure after image process method and Figure 4(b) demonstrates the reconstructed three-dimensional brain model by 92 slices that corresponded to original *in vivo *three-dimensional MRI data (Figure 4(c)).

### Monte Carlo algorithm

Photon migration in human tissue can be numerically simulated by the Monte Carlo algorithm [33-40]. The photon trajectory can be computed with the parameters for propagation governing: 1) the mean free path of a scattering or absorption event, 2) the boundary conditions - refraction and specular reflection, 3) scattering event - deflection and azimuth angles, 4) absorption event - energy loss, 5) detector location. The Monte Carlo model relies on the sampling of random variables from their probability density function. The probability density function of step size *s*_1 _is defined as:

(10) $$P\left(s_{1}\right)\;=\;\mu_{t}e^{-\mu_{t}s_{1}}$$

According to the probability density function as uniform distribution, *s*_1 _can be obtained:

(11) $$s_{1}=\frac{-\text{ln}\left(1-\xi \right)}{\mu_{t}}$$

where ξ is uniformly distributed between [0,1]. Consider a multi-layer structure that the photon may experience a free path over multiple layers before a scattering event, the counterpart of Eq. (11) becomes:

(12) $$\underset{i}{\sum }\mu_{ti}S_{i}\;=\;-\;\text{ln}\;\xi$$

where *μ_ti _*is the extinction coefficient and *s_i _*is the path length of the *i*_th _voxel. The Snell's law and Fresnel reflection formulas were applied at each boundary. The probability for the occurrence of a scattering process over the distance *d*_s _was given by

(13) p(scattering inds)=μs'ds

The probability distribution of scattering angle *θ *was assumed by Henyey and Greenstein function with the anisotropy factor *g *as

(14) $$p\left(\text{cos}\theta \right)\;=\;\frac{1\;-\;g^{2}}{4\pi {\left(1\;+\;g^{2}\;-\;2g\;\text{cos}\left(\theta \right)\right)}^{3/2}}$$

The probability distribution of azimuth angle *Ψ *was assumed to be isotropic as

(15) $$\xi \;=\;\overset{\psi }{\underset{0}{\int }}\frac{1}{2\pi }d\psi \;=\;\frac{\psi }{2\pi }$$

where *Ψ *= 2*πξ *with the uniform random number *ξ *∈ [0, 1]. At each scattering event, an individual photon packet dropped part of its power and the energy loss can be represented by

(16) $$\Delta w\;=\;w\times \frac{\mu_{a}}{\mu_{t}}$$

where *w *is the weight of the photon packet before the scattering event. In addition, the formal solution, Mie theory, describes absorption and/or scattering event with a sphere that has been available in previously study [41]. The photon-passed voxels were all recorded with temporal evolution for photon footprint tracing. Therefore, the dynamic behavior of photon migration in human brain can be manifested. Additionally, we recorded all the paths of the received photons in the simulations, the visited layers of each photon were marked. Accordingly, spatial sensitivity profiles (SSP) of adult head models were calculated from the accumulated trajectories of photons. The spatial sensitivity has been described theoretically by photon measurement density functions or sensitivity maps [39,42,43].

**Figure 5:**
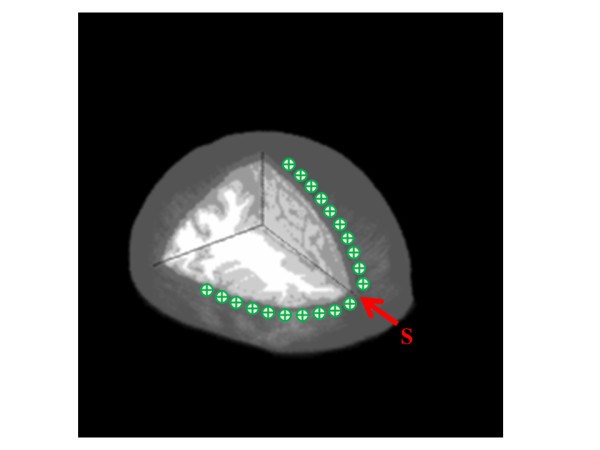
**The geometric configuration of source-detector**. The source-detector separations on human head model in simulation with transverse view and sagittal view. The separations are 1-10 cm with 1 cm step.

The source and multi-detectors arrangement on the surface with transverse view and sagittal view were applied to investigate the light propagation in human brain (shown in Figure 5). The source-detector separations in transverse view and sagittal view are 1-10 cm with 1 cm step. First, all cases were simulated at typical 800 nm wavelength illumination with the five-layer scattering/absorption coefficients 1.9/0.018 (scalp), 1.6/0.016 (skull), 0.24/0.004 (CSF), 2.2/0.036 (gray matter) and 9.1/0.014 (white matter) mm^-1 ^[16,21,23,36]. For NIRS modeling, multi-wavelength sources (690, 780, and 830 nm) were applied for illumination of the adult brain. The reduced scattering coefficient *μ*_s_', absorption coefficient *μ*_a_, scatters' radius, refractive indices of background and scatters of brain tissues are described in Table 1[35].

**Table 1:** Optical properties of brain tissues in Monte Carlo simulation

Brain tissues	*μ*_a_/*μ*_s_' at 690 nm (cm^-1 ^)	*μ*_a_/*μ*_s_' at 780 nm (cm^-1 ^)	*μ*_a_/*μ*_s_' at 830 nm (cm^-1 ^)	Anisotropy factor (g)
**Scalp**	0.159/8	0.164/7.1	0.191/6.6	0.92

**Skull**	0.101/10	0.115/9.1	0.136/8.6	0.92

**CSF**	0.004/0.1	0.017/0.1	0.026/0.1	0.92

**Gray matter**	0.178/12.5	0.170/11.6	0.186/11.1	0.92

**White matter**	0.178/12.5	0.170/11.6	0.186/11.1	0.92

**Air**	--	--	--	--

## Results

**Figure 6:**
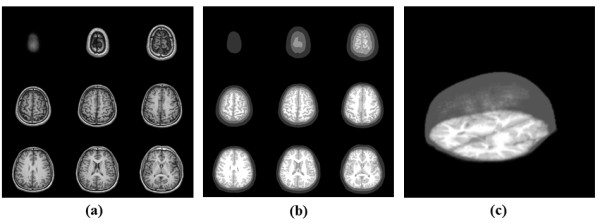
**The tomograms with different depths of the brain**. This figure shows the result of optical brain modeling from *in vivo *MRI data. (a) shows nine MRI slices of adult brain, (b) shows the processed optical model with respect to *in vivo *MRI slices, (c) shows the 3D adult brain structures of reconstructed optical models for Monte Carlo simulation.

Figure 6 shows the tomograms of the adult brain structures of *in vivo *MRI data and processed optical models for Monte Carlo simulation. The 92 two-dimensional slices were used from head top to down and then the three-dimensional images were reconstructed of both structures. The depth of head model was 9.2 cm.

**Figure 7:**
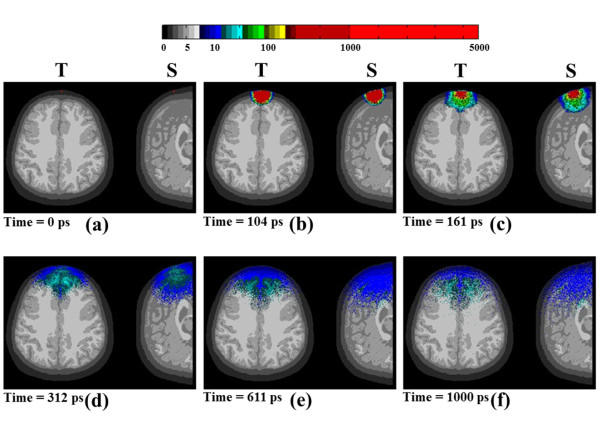
**The photon migration in the horizontal cross section**. The dynamics of photon migration in transverse and sagittal views with 800 nm light illumination: (a) at time = 0 ps, (b) at time = 104 ps, (c) at time = 161 ps, (d) at time = 312 ps, (e) at time = 611 ps, and (f) at time = 1000 ps.

The light source was located on the frontal surface at the 6 cm from the head top in transverse and sagittal view (shown in Figure 5). Based on time gating approach, the temporal responses of photon migration in human brain was made. Figure 7 shows the trajectories of all 2 × 10^7 ^photons in the transverse and sagittal cases of brain structure. Obviously, the difference patterns of photon migration can be observed. In the result, the light can reach to white matter layer (~3 cm depth) after 100 psec in transverse views. The result of simulation revealed the photon guiding effect in CSF layer. In our simulation, all photon-passed voxels were recorded for photon migration analysis. The CSF light guiding effect can be easily observed in the movie file. This paper has supplementary downloadable material available of Additional file 1 provided by the authors. This includes a multimedia MOV format movie file, which shows the dynamic photon migration with 800 nm light pulse illumination through the adult brain model. Two cross-sectional views are demonstrated as transverse and sagittal. This material is 7.65 MB in size.

**Figure 8:**
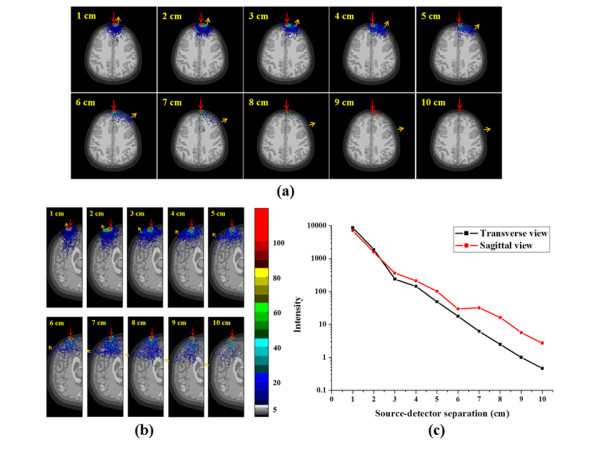
**The spatial sensitivity profile with various source-detector separations**. This figure shows the photon migration of the received photons with different distances of source-detector separation. (a) Photon trajectories via source-detector separation in transverse view, (b) photon trajectories via source-detector separation in sagittal view, and (c) the received intensity via source-detector separation.

Figure 8 demonstrates the paths of detected photons via source-detector separation in transverse and sagittal view of individualised model. Ten detectors were placed away from the light source with 1-10 cm as Figure 5. The red arrow indicates the location of light source and orange one represents each detector. The power of received light was decreasing while source-detector separation increasing. According to the result, the optimal source-detector separation was chosen between 2 and 4 cm for brain detection in this individualised model. However, Figure 8(c) indicates the longer propagation distance of diffuse photon in sagittal view because of its bigger CSF volume in interhemispheric fissure. Therefore, the behavior of light propagation in human brain strongly depends on individualised structure of brain.

**Figure 9:**
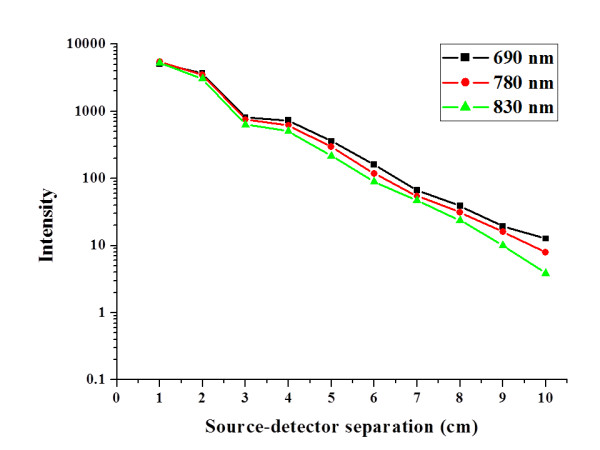
**The curve of intensity distribution with multi-wavelength**. This figure shows the multi-wavelength (690 nm, 780 nm and 830 nm) distribution of received intensity with various source-detector separations in adult brain models.

The proposed modeling method can offer multi-wavelength illumination. Figure 9 shows the curves of intensity distribution with various source-detector separations in this individualised brain model at 690 nm, 780 nm and 830 nm. Figure 9 implies that the propagated photon through brain was absorbed stronger at longer wavelength. Also, the result reveals that the change rate of detected intensity via source-detector separation can provide a quantitative analysis to evaluate the brain structure individually.

**Figure 10:**
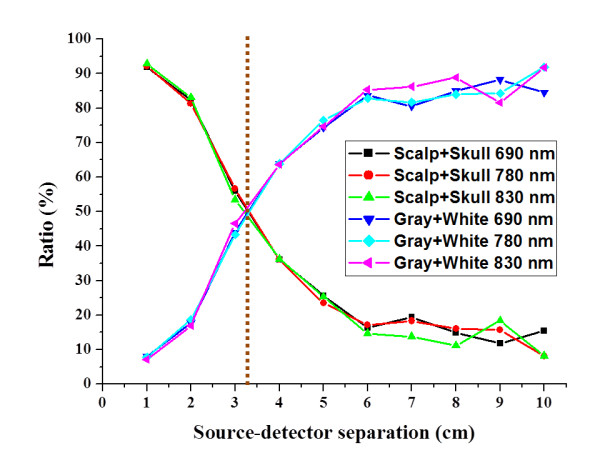
**The ratios of the backscattered intensities from different layer**. This figure shows the distributions of ratio of the received intensity from different layers of brain versus the distance of source-detector separation with multi-wavelength.

In this study, the paths of received photons from each layer were recorded. Figure 10 shows the ratios of the backscattered intensities from different layer versus the source-detector separation with multi-wavelength. Obviously, the signal from the surface (scalp and skull) layer and cerebral cortex layer were crossed at about 3.3 cm of source-detector separation. In this result of this individualised model, the backscattered light from the cerebral cortex layer is greater than 50%, while the source-detector separation exceeds the cross-point. Compare this result with Figure 9, the total received intensity was decreases strongly with the source-detector separation increasing. Hence, the source-detector separation in this individualised case was optimally set as 3.3 cm for NIRS measurement.

## Discussion

In this paper, the patient-oriented and individualized simulation for brain monitoring by using *in vivo *MRI data was proposed. An adult brain was modeled in three-dimensional time-resolved Monte Carlo simulation to investigate the structural characteristic of individualized brain. The result indicates that the three-dimensional model which faithfully represents the realistic and individualized human head from MRI data depends on image processing. In order to detect the characteristic of individualized structural with NIRS measurement, the various source-detector separations on human head were simulated dynamically with transverse and sagittal views in an adult brain model. Although the previously studies indicated the light guiding effect occurred in the CSF layer of human head. We described the Monte Carlo method that is capable of performing the penetration analysis with dynamic photon migration movies to show more clearly effects of light guiding by CSF. The penetration of 800 nm light via the CSF is remarkably clear in the Figure 7.

The CSF is a region that is filled with clear low scattering fluid in the head. In other words, the effect of both low scattering and absorption coefficients in the CSF layer reveals a strong effect on light propagation in the head. Thus, the photons propagate longer distance along the CSF layer can be observed clearly in the movie file of Figure 7, especially in sagittal view. In the Figure 8, we observed that the penetration depth in sagittal view is longer than transverse with source-detector separation from 3 cm to 10 cm, especially from 6 cm to 10 cm. In other words, the transverse cross-section contains bigger volume of gray and white matter and smaller volume of CSF than the sagittal cross-section that can observe in Figure 8. Besides, the gray and white matters generate absorption and multi-scattering that cause shallow penetration in transverse cross-section. On the contrary, the CSF layer provides low extinction of light that can help light propagation longer in sagittal cross-section. The results showed that the spatial sensitivity profile in the head formed unlike the well-known "banana" shape when the source-detector separations are less than 3 cm, which covered uniformly between the source and the detector, and covered the gray matter and even the surface of the white matter. Significant distortions were observed around the cerebral cortex folding. The spatial sensitivity profile penetrated deeper to the brain in the case of expanded CSF. Accordingly, the cerebral cortex folding geometry was suggested to significantly affect the spatial sensitivity profile in human head because it filled with CSF. This was discrepant with the previous finding by loose brain models [39,40]. However, accurate modeling of the brain structure based on image segmentation process, the effect of the sulcus (filled of CSF) on the spatial sensitivity profile was obvious. Therefore, in most studies using the brain models based on MRI data of the adult brain [13-15,17,38,39], the cerebral cortex folding in the models were suggested to be not exact and large enough to affect the spatial sensitivity profile. In the sagittal section, the photons propagate longer distance along the CSF layer can be observed clearly because the expanded interhemispheric fissure. This finding suggests that the optical method may provide not only functional signal from brain activation but also structural information of brain atrophy with the expanded CSF layer.

The multi-wavelength simulations at 690, 780, and 830 nm demonstrate nicely effects of increasing absorption with wavelength and light guiding effect through the cerebral cortex folding.

In previous studies, the source-detector separation is usually chosen between 2 and 3 cm in the previous studies. It is still a trade-off problem between the received intensity and the useful information in NIRS measurement. To our knowledge, this is the first study to provide an individualized modeling method for patient-oriented simulation with NIRS measurement. In the Figure 10, the ratio of the received intensity indicates the existence of brain activation signals from the surface of cerebral cortex (surface of gray and white matter). According to the distribution of received intensity versus source-detector separation (shown in Figure 9) and the ratio of the received intensity from different layers (shown in Figure 10), our results suggest that the optimal choice of source-detector separation for this individualized case is set as 3.3 cm. In this paper, the new contributions are stated as follows:

1. In previous studies, although the results of Monte Carlo simulation of light propagation in full segmented 3D MRI model of the human head was presented and the code was released for use by other researchers, it was an only one regular brain model. In our study, we provided an efficient and systematic modeling method of individual brain model for patient-oriented measurement and analysis. The signal-to-noise ratio evaluation and optimal choice of source-detector separation for individualized brain may provide more helpful information for NIRS systems design.

2. Li et. al. indicated that the significant characterization on the visible Chinese human model was significantly stronger than that on the MRI model [11]. Additionally, we clarified and proved that the three-dimensional model which faithfully represents the realistic human head from MRI data depends on image processing.

3. Currently, most Monte Carlo simulations have been suited to a single wavelength. However, the NIRS system usually applies multi-wavelength. In our study, we reformed the Monte Carlo simulation for multi-wavelength sources to approach the practical NIRS measurement.

## Conclusions

In conclusion, the three-dimensional time-resolved brain modeling method approaches the realistic human brain that provides useful information for NIRS systematic design and calibration for individualized case with prior MRI data. Besides, NIRS, with its advantages, could be a useful research tool for the diagnosis of patient-oriented in the near future.

## Competing interests

The authors declare that they have no competing interests.

## Authors' contributions

CC (first author) contributed in the theoretical model, proposal of the method, and writing of the manuscript. YL, CC (third author), YH and TL contributed equally in the analysis of algorithms. CS conceived of the study, and participated in its design and coordination and helped to writing the manuscript. All authors read and approved the final manuscript.

## Supplementary Material

Additional file 1 **The movies of the dynamics of photon migration in brain models**. The movie shows the dynamic photon migration with 800 nm light pulse illumination through the adult brain models. Two cross-sectional views are demonstrated as transverse and sagittal. Click here for file
